# Recovering Quality of Life (ReQoL): a new generic self-reported outcome measure for use with people experiencing mental health difficulties†

**DOI:** 10.1192/bjp.2017.10

**Published:** 2018-01

**Authors:** Anju Devianee Keetharuth, John Brazier, Janice Connell, Jakob Bue Bjorner, Jill Carlton, Elizabeth Taylor Buck, Thomas Ricketts, Kirsty McKendrick, John Browne, Tim Croudace, Michael Barkham

**Affiliations:** 1 School of Health and Related Research, University of Sheffield, Sheffield, UK; 2 Optum Patient Insights, Rhode Island, USA and University of Copenhagen, Copenhagen, Denmark; 3 School of Health and Related Research, University of Sheffield, Sheffield, UK; 4 Department of Epidemiology and Public Health, University College Cork, Cork, Ireland; 5 Dundee Centre for Health And Related Research, University of Dundee, Dundee, UK; 6 Centre for Psychological Services Research and Department of Psychology, University of Sheffield, UK

## Abstract

**Background:**

Outcome measures for mental health services need to adopt a service-user recovery focus.

**Aims:**

To develop and validate a 10- and 20-item self-report recovery-focused quality of life outcome measure named Recovering Quality of Life (ReQoL).

**Method:**

Qualitative methods for item development and initial testing, and quantitative methods for item reduction and scale construction were used. Data from >6500 service users were factor analysed and item response theory models employed to inform item selection. The measures were tested for reliability, validity and responsiveness.

**Results:**

ReQoL-10 and ReQoL-20 contain positively and negatively worded items covering seven themes: activity, hope, belonging and relationships, self-perception, well-being, autonomy, and physical health. Both versions achieved acceptable internal consistency, test–retest reliability (>0.85), known-group differences, convergence with related measures, and were responsive over time (standardised response mean (SRM) > 0.4). They performed marginally better than the Short Warwick-Edinburgh Mental Well-being Scale and markedly better than the EQ-5D.

**Conclusions:**

Both versions are appropriate for measuring service-user recovery-focused quality of life outcomes.

**Declaration of interest:**

M.B. and J.Co. were members of the research group that developed the Clinical Outcomes in Routine Evaluation (CORE) outcome measures.

There is a growing interest in using patient-reported outcome measures (PROMs) to capture the changes experienced by mental health service users. Traditionally, mental health outcomes have tended to be symptom-based rather than reflecting the process of service users’ recovery in their quality of life. Although there are measures focusing on the process of recovery,^1^ a recent review identified the need for a PROM that measures the outcomes of recovery in terms of those aspects of quality of life that matter to mental health service users.^2^ The concept of recovery for people experiencing mental health difficulties has also received greater emphasis recently, prompting demands for new measures.^3^ One influential framework identified the following components: Connectedness, Hope, Identity, Meaning and Empowerment (CHIME).^4^ In a separate study involving a systematic literature review and interviews,^5^^–^^7^ service users identified similar themes as being important to their quality of life.

Currently, existing generic PROMs used in mental health populations, for example the EQ-5D health status measure^6^^,^^8^^,^^9^ or the Short Warwick-Edinburgh Mental Well-being Scale (SWEMWBS)^10^^,^^11^ were not developed specifically for use with mental health populations. The EQ-5D has been adopted in the UK for routine outcome measurement and is preferred by the National Institute for Health and Care Excellence (NICE) to calculate quality-adjusted life-years (QALYs) for use in cost- effectiveness analyses.^12^ Although it has been shown that the EQ-5D is valid and responsive for depression, the results for anxiety disorders are less convincing.^6^^,^^13^ Research in schizophrenia,^14^ other psychotic populations,^13^^,^^15^ and bipolar found conflicting evidence on validity. For personality disorders, the EQ-5D may be suitable but lacks the content validity to fully reflect the impact of the condition.^16^ In terms of the SWEMWBS, there is limited evidence on its validity in the area of mental health.^10^^,^^11^

Guidelines published by the US Food and Drug Administration (FDA) and others on developing PROMs state that a combination of quantitative and qualitative methods should be used.^17^^,^^18^ Furthermore, the process should fully involve service users in all stages of instrument development including design, data collection, analysis and final decisions regarding content.^19^ This is not true for the generic measures mentioned above. Measures used in mental health services tend to be focused on specific symptoms such as depression (PHQ-9)^20^ or anxiety (GAD-7),^21^ both of which are used in the Improving Access to Psychological Therapies (IAPT) programme^22^ and do not meet these guidelines. Both these measures were developed on the basis of the best fit to the diagnostic criteria set out in the DSM^23^ rather than the lived experiences of service users and neither of them reflects the broader views of quality of life outcomes. The Clinical Outcomes in Routine Evaluation (CORE) measures^24^^–^^26^ tap into well-being and functioning in addition to symptoms but their development focused on input from practitioners rather than service users. What is lacking for research and clinical purposes is a short, self-report measure that is based on the outcomes service users identify as being most central to them in recovering their quality of life rather than simply reducing symptoms. To fill this gap, we report on the development and validation of a 10- and 20-item version of a user-friendly PROM named Recovering Quality of Life (ReQoL).

## Method

**Fig. 1:**
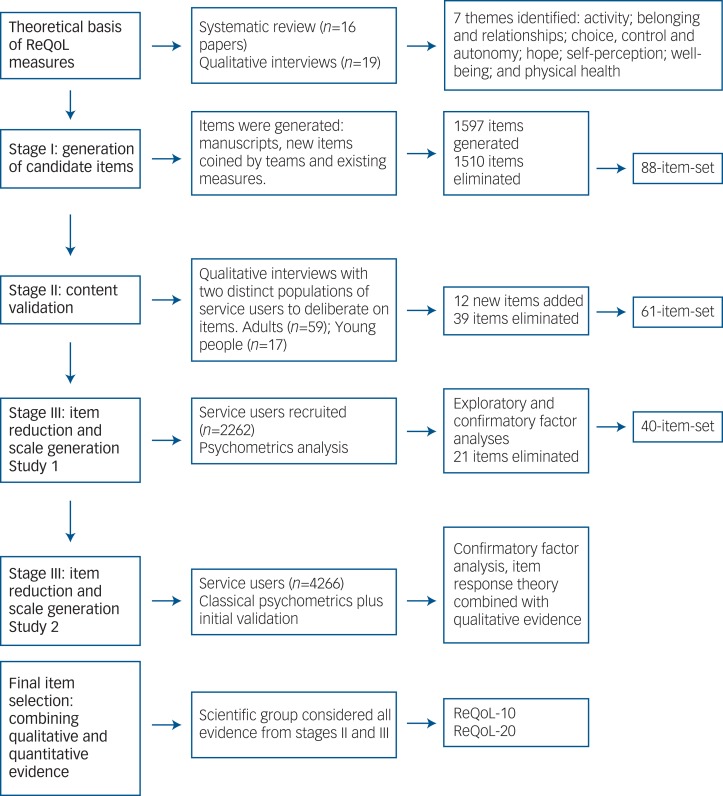
Development of the Recovering Quality of Life (ReQoL).

The ReQoL measures were developed and validated in three inter-linked stages: (a) generation of candidate items; (b) testing face and content validity of shortlisted items; and (c) psychometric evaluation. Figure 1 summarises these three stages.

Throughout the development process there was consultation with, and inputs from, four different constituencies. These comprised 33 academics (the advisory group), 32 policymakers and clinicians (the stakeholder group), six psychometricians (the psychometrics group), and six service users (the expert-user group). The final decisions were made by a fifth group (the scientific group) comprising a mix of six service users, five clinicians, five academics and two clinical academics. Ethical approval was obtained from the Edgbaston NRES Committee, West Midlands (14/WM/1062). Informed consent was obtained from all participants in the study.

### Stage I: generation of candidate items

At the start of the development process, seven themes established in prior research^5^^,^^6^ were first agreed by the scientific group in order to provide a theoretical underpinning for the measures in terms of a set of relevant concepts/constructs. The agreed themes pertaining to the concepts of both recovery and quality of life were: activity (meaningful and/or structured), hope, belonging and relationships, self-perception, well-being, autonomy, and physical health. Service users reported both positive and negative aspects of the themes (for example, hope/hopelessness), which either enhanced or depleted their quality of life.^5^^,^^7^ A pool of potential items was then generated for each of the themes from transcripts of in-depth interviews^5^ and examination of the content of 38 existing PROMs used with mental health populations. These two approaches yielded a 1597 item-set. Seven criteria adapted from the research literature (see supplementary Table DS1, available at https://doi.org/10.1192/bjp.2017.10)^27^ were applied to reduce and, if necessary, improve the pool of 1597 items through eight rounds of deliberation. We retained 88 items following consultation with the five constituency groups described above (see supplementary Fig. DS1).

### Stage II: testing face and content validity of shortlisted items

In stage II the views of 59 adult service users (aged 19–79) and 17 young people (aged 16–18) with a broad range of diagnoses were gathered on the face and content validity of the 88 items generated in stage I. Participants took part in individual interviews (*n* = 55) and focus groups (21 people in seven groups) to comment on differing subsets of the candidate items. They chose their preferred items where there were several addressing a similar subtheme, suggested new items and rephrased existing items. An iterative process was adopted in which the new and rephrased items were deliberated on in subsequent interviews ensuring that all items were checked by service users. Items that were deemed potentially distressing, judgmental, difficult to respond to, not considered relevant to everyone, and too open to different interpretations were eliminated. Details of this stage are documented elsewhere.^28^

As part of the consultation process, feedback on the items was obtained from a group of 11 clinicians working for two mental health service providers. Additionally, focus groups were carried out with 35 clinicians including staff from all the main professional groups involved in multidisciplinary mental healthcare from six different providers. Finally, a translatability assessment following established guidelines^29^ was carried out to identify potential semantic and structural issues that might be a barrier to future translations of items in the measures. The qualitative results from all the service users, clinicians and the translatability assessment were combined to further reduce the number of items to 61 (see Fig. 1).

### Stage III: item reduction and scale generation

Stage III comprised two quantitative studies to explore the dimensionality of the item-set and to inform the final item selection for the measures. Participants comprising both in-patients and out-patients were recruited from 13 and 20 secondary mental health providers in study 1 and study 2, respectively. There were also participants from: three general practices, a trial cohort in each study, and voluntary sector organisations (three and two in studies 1 and 2 respectively). In study 1, a total of 520 participants were recruited from an online panel. To maximise response rates, a combination of modes of recruitment was used. Participants were recruited face-to-face while attending services, some completed the survey by post and others online. Table 1 presents the demographics of participants from both studies. In study 1, 2262 (response rate 32%) participants completed the 61-item set at one time point only. In study 2, 4266 participants (response rate 30%) completed a reduced 40-item set (see below), of whom 953 completed a follow-up 6–12 weeks later (response rate 22%). Participants in study 2 also completed one of the following measures: EQ-5D-5L, SWEMWBS, CORE-10, PHQ-9 and GAD-7.

**Table 1:** Characteristics of the samples recruited in the psychometric testing stages

	Stage III	
	Study 1 (*n* = 2262)	Study 2 (*n* = 4266)
Age categories, years: *n* (%)		
16–25	261 (12)	441 (10)
26–64	1541 (68)	2879 (67)
≥65	390 (17)	681 (16)
Missing	687 (3)	265 (6)
Ethnicity, *n* (%)		
White	1932 (85)	3649 (86)
Black and minority ethnic	296 (13)	384 (9)
Missing	32 (1)	233 (5)
Diagnoses, *n* (%)		
Common mental health disorders	794 (35)	1423 (33)
Schizophrenia	213 (9)	421 (10)
Other psychotic disorders	116 (5)	234 (5)
Bipolar	201 (9)	411 (10)
Personality disorder	106 (5)	238 (6)
Others	239 (11)	252 (6)
Missing	593 (26)	1287 (30)
Recruitment setting, *n* (%)		
General practices	145 (6)	1146 (27)
IAPT	–^a^	261 (6)
Secondary care, out-patients	1288 (57)	1976 (46)
Secondary care, in-patients	64 (3)	563 (13)
Community	765^b^ (34)	310 (7)
Life satisfaction,^c^ mean (s.d.)	5.4 (2.7)	5.3 (2.8)

a. In study 1, participants from Improving Access to Psychological Therapies (IAPT) had been recruited but classified as secondary care, out-patients.

b. This includes participants recruited from the: online panels, trial cohort and voluntary organisations.

c. How satisfied are you with your life nowadays? Score 0, not satisfied at all to 10, extremely satisfied.

### Factor analyses (studies 1 and 2)

Confirmatory factor analysis (CFA) and exploratory factor analysis (EFA) (Geomin rotation) were carried out in both studies in order to establish dimensionality. Items concerning a physical health theme were excluded from the factor analyses as physical health was deemed *a priori* and conceptually to be a different construct. As the item responses were captured on a five-point Likert scale, the variables were treated as ordinal categorical. The factor analyses were carried out in Mplus 7.4.^30^ Model fit was assessed by the root mean square error of approximation (RMSEA) and the Comparative Fit Index (CFI). For the RMSEA and CFI, a value of ≤0.08 and >0.95 was assumed to provide a good fit, respectively.

### Item response theory (IRT) analyses (study 2)

Graded response models (GRM)^31^ were used for all analyses to inform item selection. Model fit was evaluated by the sum-score based item fit statistic (S-G^2^).^32^ Since the S-G^2^ statistic is calculated for each item, the approach may lead to spurious results in cases of large numbers of items. To counter this problem, study 2 was divided into four data-sets (*n* > 1000 each). A sample size of a minimum 1000 was considered sufficient to identify relevant misfit. Only items with misfit (*P* < 0.05) in 3–4 data-sets were considered misfitting. After fitting the IRT models, item and test information functions were examined. Information functions indicate the precision of measurement for people at different levels of severity on the latent scale and are dependent on the item parameters. All IRT analyses used IRTPRO 3.0.^33^

Differential item function (DIF) with regards to age, gender, ethnicity and diagnosis was evaluated through ordinal logistic regression models.^34^ Significant DIF was assessed through dual criteria of statistical significance and a difference in explained variance (Nagelkerke pseudo *R*^2^) larger than 2%.^35^

### Final item selection: synthesising qualitative and quantitative evidence

For each item, the quantitative and qualitative evidence was synthesised to ensure that the best and most acceptable items for the two versions of ReQoL were chosen. An initial item was first chosen for each theme followed by a decision on whether a second item was needed to cover all aspects of the theme.

### Scoring of the 40-item set

Two methods of scoring were compared. From the IRT analyses, expected *a posteriori* (EAP) scores were calculated that estimate the expected value of the probability distribution of latent trait scores.^36^^,^^37^ IRT scores at baseline and changes in scores were compared with summative scores for the whole sample and also for primary *v.* secondary care participants. To aid direct comparisons, ReQoL-20 scores were halved so that both versions were within the range 0–40. ReQoL-10 scores were calculated if no more than one item had a missing response. The ReQoL-20 scores were calculated if no more than two items were missing. In both cases, the mean value of the other responses was used to impute the score for the missing item or items.

### Reliability

For the reliability assessment, a sample comprising both patients and members of the general population was recruited from an online panel through a market research company. A total of 2000 members of the general public and 800 patients were recruited. The general population sample recruited was representative of the UK general population based on age, gender (46% male, 54% female), ethnicity (92% white) and geography. A total of 74% (*n* = 595) of the patient population reported experiencing common mental health disorders, either depression only, anxiety only or both. Among the patient sample, 78% reported very poor to fair mental health compared with 26% in the general population sample. The majority of respondents were female (61%) and 97% of the population were white. Half of the participants in each group completed ReQoL-10 and the other half completed ReQoL-20 in their final formats. A subset of each sample was asked to complete ReQoL-10 and ReQoL-20 approximately 2 weeks apart. The follow-up questionnaires were completed by participants from the patient (*n* = 141) and general population (*n* = 350) samples to examine test and retest reliability. Reliability was assessed by the intraclass coefficient (ICC) where an ICC >0.70 would indicate very good test–retest reliability.

Internal reliability was assessed using Cronbach alpha to assess the extent to which the items were interrelated. Coefficients above 0.7 are acceptable, above 0.8 are good and above 0.9 are excellent but above 0.94 suggests potential redundancy.^38^

### Construct validity

Convergent validity between ReQoL and two other measures, SWEMWBS and CORE-10, was assessed using Pearson's product moment correlation coefficients. Strong correlations were expected between the ReQoL measures, SWEMWBS and CORE-10 as they reflect common mental health-related aspects of quality of life. Correlations are considered strong if scores are ≥0.7.^39^

Known-group validity was examined in terms of whether the ReQoL measures were able to discriminate between the general population and those people with a variety of specific conditions (i.e. depression, anxiety, schizophrenia, bipolar, personality disorder and other conditions). For those with anxiety or depression the known-group validity was also assessed using GAD-7 and PHQ-9 cut-off scores as well as CORE-10 clinical cut-off points (where a score of ≥10 and ≥ 11 for PHQ-9 and CORE-10 respectively indicate clinical concerns). Although GAD-7 and PHQ-9 do not measure aspects of quality of life, they are thought to define broad groups expected to generate different quality of life scores. We also investigated known-group validity by using a self-reported global assessment of health and mental health. The five original categories were collapsed into binary categories of poor *v.* good health. Differences were quantified using standardised effect sizes (SES) across severity subgroups calculated as the difference in mean scores between groups divided by the standard deviation of the milder of the two subgroups. SES expressed as Cohen's *d* of 0.2 are normally considered small, 0.5 moderate, and 0.8 large.^39^

### Responsiveness

We measured responsiveness in two ways. First, we examined the numbers of people with either the lowest possible score or highest possible score since they have an impact on the ability of the ReQoL measures to detect deterioration or improvements, respectively. Second, we used the sensitivity to apparent changes in quality of life. In the absence of an objective measure of change, we used the responses of people reporting mental health problems to a quality of life transition item that asked whether they thought their quality of life had stayed the same, improved (somewhat or a lot) or worsened (somewhat or a lot) since they last completed the questionnaire between 6 and 12 weeks ago. Responsiveness for ReQoL, SWEMWBS and EQ-5D was assessed using the standardised response mean (SRM) statistic, calculated by dividing the mean change on the measure by the standard deviation of the change. Similar cut-offs as for SES above were used.

## Results

### Factor analyses

In the initial CFA, the six mental health factors did not provide a satisfactory model and the factors were strongly correlated. The results from the EFA of the mental health items suggested a two-factor solution. All the negatively worded items (*n* = 34) loaded on the first factor and all the positively worded items (*n* = 23) loaded on the second factor. The correlation between the two factors was 0.8. A bifactor model comprising a global factor and two local factors of negative and positive affects yielded a slightly superior fit. The factor loadings on the negative and positive factors were considerably smaller than the loadings on the global factor, thereby supporting an essentially unidimensional model. Detailed psychometrics results are presented elsewhere.^28^ Redundancy found in the factor analysis results in study 1 were combined with the qualitative evidence on the items from stage II in order to reduce the item-set from 61 to 40 items (Fig. 1). This 40-item-set comprising 39 mental health items and one physical item was retained for study 2. Similar factor analyses results were obtained in study 2.

In the IRT analyses conducted in study 2, two items were found to be misfitting. The marginal reliability for response pattern scores of the 39 items was 0.98. Although IRT scores and sum scores were strongly correlated (*r* = 0.98, supplementary Fig. DS4) both for baseline/initial assessment and repeated/follow-up ReQoL, there were noticeable differences for some participants. The correlation between change in sum scores and change in EAP scores was 0.95. IRT scoring did not provide any benefit in terms of yielding more statistical power when scores for primary and secondary care service users were compared.

The selection of the additional 10 items to constitute the 20-item version followed a similar process. ReQoL-20 items were chosen to provide more item information on important subthemes (such as sleep, concentration and control of life). This makes little difference to the overall psychometric performance.^28^

### Psychometric evaluation

#### Distribution of scores

Missing data did not exceed 5% for any of the items (including the 40-item set) and no obvious ceiling or floor effects were observed (supplementary Table DS7a). Missing data ranged between 3 and 4% for all mental health items of the ReQoL (supplementary Table DS2) and scores could not be calculated for 5% of the sample. Imputation for missing data was performed for 5% and 11% of the sample to obtain ReQoL-10 and ReQoL-20 scores, respectively. Missing data rates were less than 5% for the comparator measures.

The means and standard deviations for ReQoL-10 and ReQoL-20 at baseline were 21.99 (s.d. = 10.26) and 21.63 (s.d. = 9.97), respectively (supplementary Table DS5). All response options for both ReQoL measures were endorsed and the ReQoL scores covered the full range of the 0–40 scale (supplementary Fig. DS2). The overall score distributions were well distributed across the score range, although there were some spikes and noticeably smaller numbers at the lower end of the scales (i.e. low quality of life). The means and standard deviations for the three comparator measures were: EQ-5D (*n* = 1592), mean 0.75 (s.d. = 0.25); summative and transformed SWEMWBS (*n* = 1103) scores, mean 23.14 (s.d. = 6.80) and 21.71 (s.d. = 5.85), respectively; and CORE-10 (*n* = 216), mean 17.79 (s.d. = 10.94) (supplementary Table DS5).

### Reliability

The ICC for the ReQoL-10 measure for both the general population sample (*n* = 488) and the patient sample (*n* = 279) reporting the same general mental health at both administrations was 0.85 (*P* < 0.01). For ReQoL-20 the ICCs for the patient sample (*n* = 100) and the general population sample (*n* = 249) were 0.90 and 0.87, respectively. Cronbach alphas for the embedded ReQoL-10 and ReQoL-20 items in study 2 were 0.92 and 0.96, respectively. For the online samples, the equivalent alphas were 0.87 and 0.93 for ReQOL-10 and ReQoL-20, respectively.

### Convergent validity

The correlations of both ReQoL measures with the summative scores of the SWEMWBS and CORE-10 were above 0.80 across four main diagnostic groups and 0.90 or more for the pooled data-set suggesting a strong level of convergence (Table 2). The ReQoL-20 correlations were very similar to those of the ReQoL-10, although overall slightly higher. All correlations were significant (*P* < 0.01) and in the correct direction. The correlation between the ReQoL-10 and ReQoL-20 was 0.98. See supplementary Fig. DS3 for further details.

**Table 2:** Convergence by condition of Recovering Quality of Life (ReQoL) measures with other measures^a^

	All mental health conditions		Depression and anxiety		Schizophrenia		Bipolar		Personality disorder	
	*n*	*r*	*n*	*r*	*n*	*r*	*n*	*r*	*n*	*r*
*ReQoL-10 score with other measures*										
SWEMWBS	1050		383		52		103		46	
Total score		0.90		0.90		0.84		0.92		0.92
Rasch score		0.86		0.87		0.82		0.89		0.90
CORE-10	211	−0.88	55	−0.90	55	−0.76	25	−0.89	19	−0.89
*ReQoL-20 score with other measures*										
REQOL-10		0.98	1470	0.98	517	0.96	402	0.97	233	0.97
SWEMWBS	1050		383		52		103		46	
Total score		0.90		0.90		0.81		0.93		0.91
Rasch score		0.87		0.87		0.79		0.90		0.90
CORE-10	211	−0.93	55	−0.92	55	−0.87	25	−0.95	19	−0.96

SWEMWBS, Short Warwick-Edinburgh Mental Well-being Scale; CORE, Clinical Outcomes in Routine Evaluation.

a. All the correlation coefficients (*r*) are product moment correlations and significant at 1%.

### Known-group validity

Table 3 presents the means, standard deviations and SESs for the ReQoL-10 and ReQoL-20. The ReQoL scores for the online general population sample were significantly higher (i.e., better quality of life) compared with the six diagnostic groups of depression, anxiety, schizophrenia, bipolar, personality, and other diagnoses as broadly defined by ICD-10 codes.^40^ As shown in Table 3, the SESs show the differences were moderate for schizophrenia and other psychotic disorders and large for common mental health disorders, bipolar, personality and other mental health disorders. The SESs for ReQoL-20 were marginally larger than those for ReQoL-10. ReQoL scores distinguished between thresholds defined by the PHQ-9, GAD-7 and CORE-10. The largest SES was observed with CORE-10 cut-off and the lowest with GAD-7 score.

**Table 3:** Known-group validity for the Recovering Quality of Life (ReQoL) measures

		ReQoL-10		ReQoL-20	
	*n*	Score, mean (s.d.)	SES	Score, mean (s.d.)	SES
General population *v.* patient population			0.93		1.05
General population	1671	28.48 (6.96)		28.56 (6.57)	
Patient population	4037	21.99 (10.26)		21.63 (9.97)	
Using self-reported global assessment of mental health, good *v.* poor			1.68		1.72
Good	2633	26.56 (8.40)		26.17 (8.10)	
Poor	1223	12.46 (6.61)		12.20 (6.16)	
Comparing general population with those who self-reported the following conditions					
Common mental health disorders	1470	20.33 (9.74)	1.17	19.82 (9.31)	1.33
Schizophrenia and psychotic disorders	516	23.76 (8.89)	0.68	23.75 (8.79)	0.73
Bipolar disorder	396	21.52 (10.04)	1.00	21.29 (9.74)	1.11
Personality disorder	232	14.63 (8.41)	1.99	14.30 (8.17)	2.17
Other mental health disorders	252	18.58 (9.73)	1.42	17.87 (8.99)	1.63
*Comparing by clinical cut-offs used in clinical practice*					
PHQ-9 clinical *v.* non-clinical score			1.70		1.85
Clinical (score ≥10)	419	15.73 (7.53)		15.23 (7.08)	
Non-clinical	227	27.37 (6.83)		27.38 (6.57)	
GAD-7 clinical *v.* non-clinical score			1.03		1.21
Clinical (score ≥8)	202	14.14 (7.35)		13.22 (6.70)	
Non-clinical	318	23.02 (8.63)		23.10 (8.18)	
CORE-10 clinical *v.* non-clinical score			2.37		2.96
Clinical (score ≥11)	150	15.08 (8.02)		14.85 (7.50)	
Non-clinical	66	30.73 (6.61)		31.14 (5.51)	

SES, standardised effect size.

*P*-values are all <0.001.

The known-group differences analyses were repeated with the samples of participants who completed ReQoL and SWEMWBS and those who completed ReQoL-10 and EQ-5D (Table 4). The paired results comparing ReQoL-10 scores and SWEMWBS summative scores revealed higher SESs for ReQoL-10 in general. When comparing ReQoL-10 scores with the transformed SWEMWBS scores, similar results were observed. The head-to-head comparison between ReQoL-10 and EQ-5D found the SESs to be markedly higher for ReQoL-10 (see supplementary Table DS6).

**Table 4:** Comparing known-group validity of Recovering Quality of Life (ReQoL-10), Short Warwick-Edinburgh Mental Well-being Scale (SWEMWBS) and EQ-5D in same samples

	ReQoL-10 and SWEMWBS, summative score			ReQoL-10 and EQ-5D, summative score		
	*n*	ReQoL-10 SES	SWEMWBS SES	*n*	ReQoL-10 SES	EQ-5D SES
General population *v.* patient population	1007	0.56	0.48^a^	1513	0.64	0.64^b^
Using self-reported global assessment of mental health, good *v.* poor		1.83	1.62		1.90	1.63
Good	751			1151		
Poor	205			321		
Comparing general population with those who self-reported the following conditions						
Common mental health disorders	371	0.78	0.66	530	0.92	0.68
Schizophrenia and psychotic disorders	52	0.76	0.63	190	0.56	0.44
Bipolar disorder	98	0.91	0.75	97	0.77	0.64
Personality disorder	46	2.09	1.89	59	1.83	1.15
Other mental health disorders	–^c^	–^c^	–^c^	89	1.10	0.78

SES, standardised effect size.

a. This is the SWEMWBS transformed score as the norms for the general population norms are only provided for the transformed scores from: http://www2.warwick.ac.uk/fac/med/research/platform/wemwbs/researchers/interpretations/wemwbs_population_norms_in_health_survey_for_england_data_2011.pdf.

b. The EQ-5D norms have been provided by Devlin *et al*.

c. No data as *n* was low.

*P*-values are all <0.001.

### Responsiveness

Scores improved on all instruments between administrations. For the 953 participants at follow-up, the means and standard deviations for ReQoL-10 and ReQoL-20 were 24.18 (s.d. = 10.08) and 24.28 (s.d. = 9.78), respectively. The proportions of responses at the worst scores were below 1% and less than 5% at the best level at both baseline and follow-up. The SRMs for the ReQoL items were moderate for those reporting improvements in their health and those reporting deteriorations and <0.2 for those reporting their health had remained the same (Table 5).

**Table 5:** Responsiveness to change

	Health stayed the same			Health improved^a^			Health worsened^a^		
	*n*	Mean (s.d.)	SRM	*n*	Mean (s.d.)	SRM	*n*	Mean (s.d.)	SRM
Comparing ReQoL and SWEMWBS									
ReQoL-10	272	−0.80 (5.12)	−0.16	131	2.31 (6.04)	0.38	86	−3.61 (5.67)	−0.64
ReQoL-20	272	−0.49 (4.54)	−0.11	131	2.47 (5.22)	0.47	86	−2.92 (5.33)	−0.55
SWEMWBS^b^	272	−0.15 (4.05)	−0.04	131	1.60 (3.58)	0.45	86	−1.91 (3.82)	−0.50
SWEMWBS^c^	272	−0.15 (3.86)	−0.04	131	1.27 (3.03)	0.42	86	−1.52 (3.19)	−0.48
Comparing ReQoL and EQ-5D									
ReQoL-10	123	0.50 (6.59)	0.08	69	3.24 (8.35)	0.39	50	−3.82 (6.12)	−0.62
ReQoL-20	123	0.34 (5.94)	0.06	69	3.59 (7.65)	0.47	50	−3.47 (5.22)	−0.67
EQ-5D	123	0.17 (0.83)	0.08	69	0.82 (0.18)	0.07	50	−0.04 (0.16)	−0.25

SRM, standardised response mean; ReQoL, Recovering Quality of Life; SWEMWBS, Short Warwick-Edinburgh Mental Well-being Scale.

a. Participants self-reported using a global assessment question at follow-up; and this combines the categories ‘somewhat’ and ‘a lot’.

b. SWEMWBS – summative score.

c. SWEMWBS – transformed Rasch score.

Overall SRMs between groups were similar in magnitude for SWEMWBS and for both ReQoL versions. For patients reporting an improvement in health, the SWEMWBS SRMs were moderate in size and lay between those for the ReQoL-10 and ReQoL-20. The SRMs were marginally larger for the ReQoL instruments in those who reported their health had worsened. In contrast, the SRMs for EQ-5D were small according to Cohen's criteria and less than half those for the ReQoL versions in both groups of patients reporting change.

## Discussion

This paper has reported on the process of developing and validating two versions – ReQoL-10 and ReQol-20 – of a new measure capable of capturing service users’ recovery in their quality of life (http://innovation.ox.ac.uk/outcome-measures/recovering-quality-life-reqol-questionnaire/). The bifactor CFA model provided a good fit to the data supporting the unidimensionality of the scale. Results showed good internal reliability and test–retest reliability. Construct validity was supported by strong convergence between the ReQoL measures and the SWEMWBS. The ReQoL measures were able to distinguish between the general population and a patient population, those with four mental health conditions and between those reporting good and poor mental health. Both ReQoL measures were able to detect changes when a change in mental health was reported. The SESs and SRM for ReQoL-10 and ReQoL-20 were generally higher than for SWEMWBS and markedly better than EQ-5D.

The development of ReQoL was based on service users’ views throughout. Over 6450 service users were involved as participants in the research. Service-user involvement was not limited to being participants but importantly some were part of the research team and the decision-making process of the research. The involvement of service users is not only important for the face validity of the measures but also because of the long-standing recognition that their perspectives differ significantly from those of academics and clinicians.^19^ This paper illustrates the collaborative manner with which ReQoL was developed with service users and key stakeholders. The development process was transparent and inclusive, harnessing expertise from a range of contributors.

The ReQoL measures offer a number of important advantages over existing measures. They are the only ones known to the authors that have been built around the themes of recovery identified by Leamy *et al*.^4^ In addition, the measures contain a mixture of positive and negative items, a crucial element as people with mental health difficulties identified issues that both enhanced or depleted their quality of life. The presence of negative aspects increases the relevance of ReQoL as a PROM of recovery in mental health populations.^41^ ReQoL should offer significant advantages compared with generic measures such as the EQ-5D and SWEMWBS that were not developed in conjunction with mental health service users, as well as measures based on symptoms from one disorder, such as the PHQ-9, which is commonly used in clinics but does not reflect the broader concerns of many service users beyond depressive symptomatology.

Previous work showed that EQ-5D was not suitable for use in economic evaluation of interventions in many areas of mental health.^6^^,^^8^^,^^9^ Our findings show the ReQoL measures provide a more sensitive and responsive measure than the EQ-5D. The SRM for EQ-5D for participants reporting either an improvement or deterioration in health was 0.07 and 0.25, respectively whereas the SRMs using ReQoL-10 were 0.39 and 0.62. This will have perverse implications when using the EQ-5D to measure health benefits from a mental health intervention and may be disadvantageous in terms of resources allocated to mental health services.

The findings of little difference between ReQoL-10 and ReQol-20 in terms of reliability and validity are not surprising given that the ReQoL-10 items are contained in the 20-item version. Although the alpha of 0.96 for the ReQoL-20 suggests the presence of redundant items, all 20 items were retained in order to provide a fuller battery of items either for research studies or in order to provide a more rounded assessment in clinical settings. ReQoL-20 can be used to support more in-depth conversations between clinicians and service users about which areas service users need most support with and to help clinicians and service users to understand progress during an intervention.

Both the ReQoL-10 and ReQoL-20 can be used in routine practice or research settings. They are short, easy to use for self-completion and only take a few minutes to complete. An overall index score for ReQoL-10 and ReQoL-20 can be calculated by summing the item response numbers. The positively and negatively worded items score from zero to four and four to zero, respectively where zero on the scale represents the poorest quality of life and four the highest. In theory, the IRT score is the best available estimate of the true score. However, it comes with a price of complexity in the scoring procedure. As a result, the summative score is recommended.

### Limitations

There are several limitations to this study. Respondents were not randomly selected and may not be representative of the population experiencing mental health difficulties. In the absence of a gold standard in this field, we had to rely on indirect methods of construct validity and responsiveness to provide evidence to support the properties of the measures. We have used crude measures of known-group validity as they were the only ones that could feasibly be collected during the study. Furthermore, they were dependent on self-report because of the absence of diagnostic data. The validation results presented were assessed on the embedded ReQoL-10 and ReQoL-20 items contained in the 40-item data-set. However, we would not expect the results of the validation of the final measures to be different. Ideally, participants would have been randomly assigned one of the second measures. However, this was not practical and instead all participants recruited from any one organisation completed the same second measure. Given the large number of participants recruited across several organisations, we do not expect that this had an impact on the results. The measures need to be validated with different ethnic groups and languages. Further independent validation using the final versions of the instruments rather than the embedded forms used here is required.

### Further research

In summary, the ReQoL measures add important information to what is traditionally collected in mental health. They have excellent face and content validity and desirable properties in terms of reliability, construct validity and responsiveness. The measures also have excellent acceptability and feasibility in clinical practice. Further work will involve investigating practical issues pertaining to implementation of the ReQoL measures and interpretation of results. Future research is planned to estimate preference weights for calculating QALYs from the ReQoL-10 and -20 for use in cost-utility analyses.

## Supplementary material

For supplementary material accompanying this paper visit https://doi.org/10.1192/bjp.2017.10. click here to view supplementary material
