# Supplementary material for: Recovering Quality of Life (ReQoL): a new generic self-reported outcome measure for use with people experiencing mental health difficulties
Source: Br J Psychiatry. 2018 Jan;212(1):42–9. doi: 10.1192/bjp.2017.10 (PMC6457165; doi:10.1192/bjp.2017.10)
Supplement: Supplementary file 1 [file S0007125017000101sup001.pdf]

Table DS1 List of criteria used in Stage I of the development process, adapted from Streiner et al.[1]

1.	Reading Level	Rule of thumb: reading skills should not exceed those of a 12 year old
2.	Ambiguity	Poorly worded items<br>Even straightforward items may pose a problem if not applicable, e.g. I like my spouse<br>is problematic if someone does not a spouse
3.	Double –barrelled question	This is where two or more questions are asked at the same time and the answers for each may be different. This may also be where two different concepts are compounded e.g. anxiety and depression
4.	Jargon	The vocabulary should not be technical and should be part of everyday vocabulary.
5.	Value-laden words	Judgmental statements may prejudice the respondent and should therefore be avoided<br>(e.g. having more social contact may not be seen to be better by everyone)
6.	Positive and negative wording	Negatively worded items should be avoided e.g. it is better to have the item ' I feel ill most of the time' compared with 'I rarely feel well'
7.	Length of items	Should be as short as possible but not too short that it loses comprehensibility

In addition to the above criteria, the ReQoL research team used the following:

8.	Too specific to a lifestyle or a diagnosis	e.g. referring to employment when many respondents may not work<br>e.g. hearing voices is too specific
9.	Makes comparisons over time	e.g. the phrase 'compared to usual' in an item
10.	Does not lend itself to change over time	e.g. character traits of people or circumstances that NHS services cannot change

[1] Streiner, David L., and Geoffrey R. Norman. *Health measurement scales: a practical guide to their development and use*. Oxford Medical Publications, 1989.

Figure DS1 Stage I Generation of candidate items: Item reduction flow chart

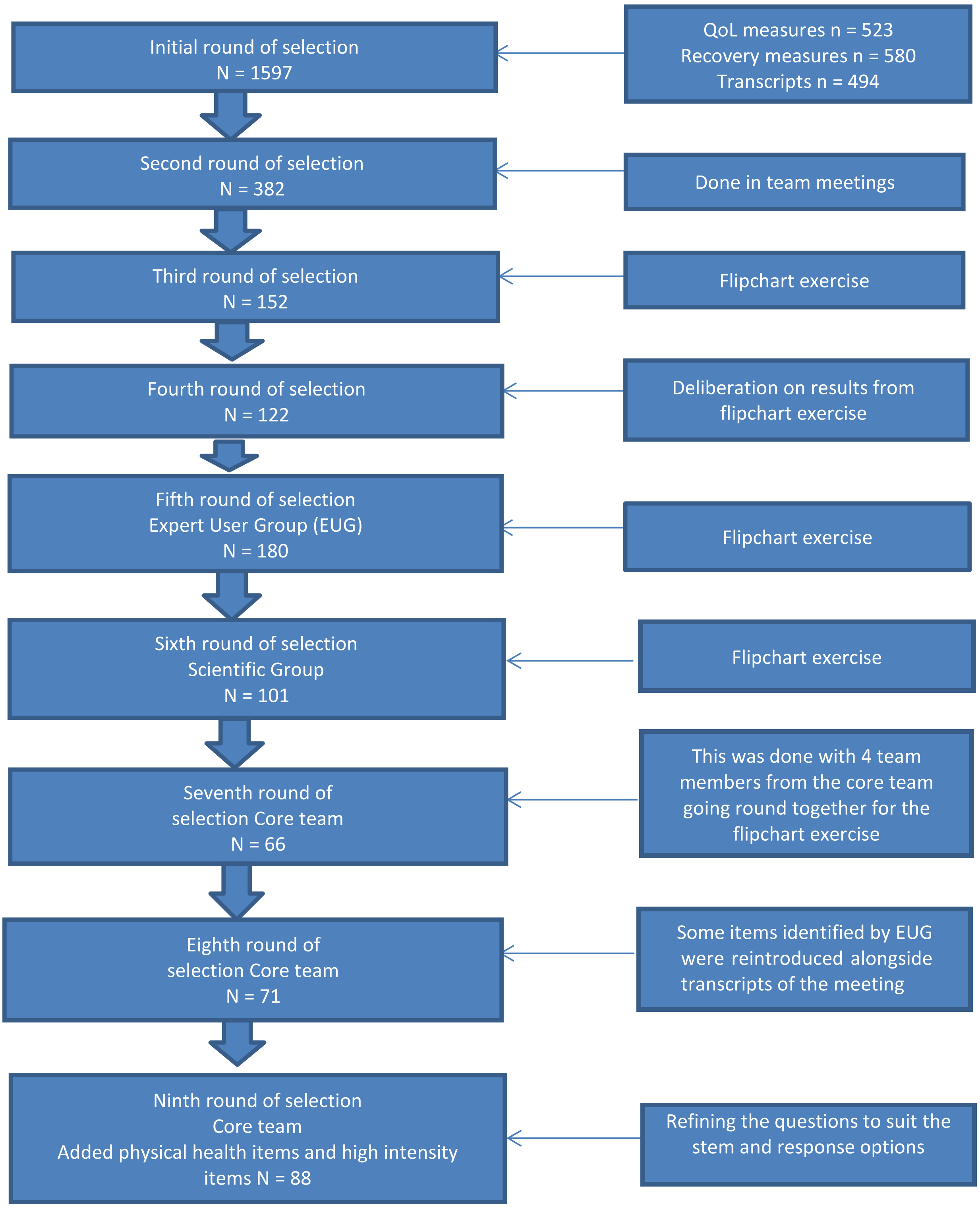

The initial selection of items was done by two researchers from the core team. The second, third and fourth rounds of selection were performed by the core team in a series of meetings. During the ‘flipchart exercise’, items were written on post-it notes on flipcharts grouped by themes and sub-themes. Members went round the room either alone or in small groups and put a sticker next to their preferred items. Votes for each item were counted and were used to inform item selection.

Table DS2 Missing data of the 40-item set by item under each theme

Theme	Item	Total sample n = 4266	
Activity	I found it difficult to get started with everyday tasks	139	3%
	I did things I found rewarding	166	4%
	I neglected myself	188	4%
	I avoided things I needed to do	173	4%
	I enjoyed what I did	164	4%
Belonging and Relationships	People around me caused me distress	145	3%
	I felt lonely	161	4%
	I felt able to trust others	171	4%
	I felt people did not want to be around me	166	4%
	I thought people cared about me	169	4%
Choice, Control and Autonomy	I could do the things I wanted to do	161	4%
	I felt overwhelmed by my problems	166	4%
	I had the opportunity to do the things I wanted	151	4%
	I felt unable to cope	175	4%
	I felt in control of my life	180	4%
Hope	I felt hopeful about my future	181	4%
	I felt hopeless	162	4%
	Everything in my life felt bad	162	4%
	I thought my life was not worth living	152	4%
Self-perception	I felt like a failure	157	4%
	I felt confident in myself	163	4%
	I felt at ease with who I am	162	4%
	I valued myself as a person	140	3%
	I disliked myself	182	4%
Wellbeing	I felt calm	137	3%
	I felt miserable	143	3%
	I felt safe	150	4%
	I was disturbed by unwanted thoughts and feelings	155	4%
	I felt irritated	165	4%
	I felt angry	154	4%
	I felt relaxed	177	4%
	I felt terrified	179	4%
	I felt everything was an effort	166	4%
	I felt panic	159	4%
	I felt happy	174	4%
	I found it hard to concentrate	159	4%
	I worried too much	161	4%
	I felt anxious	181	4%
	I had problems with my sleep	149	3%
Physical health		299	7%§

§This was higher than the rest due to the presentation of the question in the survey booklet.

Table DS3 Endorsement frequency (Study 2: n = 4266)

Item description	Levels				
	**1**	**2**	**3**	**4**	**5**
I found it difficult to get started with everyday tasks	589	853	1051	889	745
	14%	*21%*	*25%*	*22%*	*18%*
I felt able to trust others	466	822	991	893	923
	11%	*20%*	*24%*	*22%*	*23%*
I felt unable to cope	481	655	850	825	1,280
	12%	*16%*	*21%*	*20%*	*31%*
I could do the things I wanted to do	410	988	1,168	703	836
	10%	*24%*	*28%*	*17%*	*20%*
I felt happy	583	1,020	1,110	751	628
	14%	*25%*	*27%*	*18%*	*15%*
I thought my life was not worth living	381	446	573	610	2,104
	9%	*11%*	*14%*	*15%*	*51%*
I enjoyed what I did	452	834	1,234	752	830
	11%	*20%*	*30%*	*18%*	*20%*
I felt hopeful about my future	713	948	1,029	668	727
	17%	*23%*	*25%*	*16%*	*18%*
I felt lonely	623	699	807	777	1,199
	15%	*17%*	*20%*	*19%*	*29%*
I felt confident in myself	826	974	982	617	704
	20%	*24%*	*24%*	*15%*	*17%*
I did things I found rewarding	576	962	1198	781	583
	14%	*23%*	*29%*	*19%*	*14%*
I avoided things I needed to do	566	810	984	834	899
	14%	*20%*	*24%*	*20%*	*22%*
I felt irritated	483	895	1,080	983	660
	12%	*22%*	*26%*	*24%*	*16%*
I felt like a failure	686	649	717	709	1,348
	17%	*16%*	*17%*	*17%*	*33%*
I felt in control of my life	803	957	903	642	781
	20%	*23%*	*22%*	*16%*	*19%*
I felt terrified	241	377	630	655	2,171
	6%	*9%*	*15%*	*16%*	*53%*
I felt anxious	868	914	824	801	678
	21%	*22%*	*20%*	*20%*	*17%*
I had problems with my sleep	1,080	766	715	716	840
	26%	*19%*	*17%*	*17%*	*20%*
I felt calm	381	964	1,256	792	736
	9%	*23%*	*30%*	*19%*	*18%*
I found it hard to concentrate	778	877	965	842	645
	19%	*21%*	*24%*	*21%*	*16%*

Table DS4: Characteristics of the online samples for reliability

		Patients (n = 800)		General population (n = 2000)	
		Mean	SD %	Mean N	SD %
Age groups in years	18 to 24	25	3.1	223	11.2
	25 to 34	108	13.6	343	17.5
	35 to 44	147	18.4	334	16.7
	45 to 54	234	29.2	371	18.6
	55 to 64	273	34.1	296	14.8
	65 and over	13	1.6	433	21.7
Life satisfaction score	Score 0 to 10 (10 highest)	4.6	2.4	6.7	2.2
Gender	Male	311	38.9	927	46.4
	Female	489	61.1	1073	53.6
Marital Status	Single	259	32.4	560	28.0
	Married / Partner	398	49.8	1203	60.2
	Separated / Divorced	118	14.7	160	8.0
	Widowed	23	2.9	72	3.6
	Prefer not to say	2	0.2	5	0.2
Ethnicity	White	777	97.1	1833	91.7
	Non white	23	2.9		
Degree	Yes	313	39.1	996	49.8
	No	487	60.9	1004	50.2
Main activity	In employment or self-employment	332	41.5	1063	53.2
	Retired	86	10.7	507	23.3
	Housework	95	11.9	162	8.1
	Student	19	2.4	101	5.1
	Unemployed	268	33.5	167	8.3
General physical health	Excellent	25	3.1	246	12.3
	Good	210	26.2	965	48.2
	Fair	303	37.9	566	28.3
	Poor	206	25.8	189	9.5
	Very poor	56	7.0	34	1.7
General mental health	Excellent	28	3.5	628	31.4
	Good	145	18.1	852	42.6
	Fair	357	44.6	407	20.3
	Poor	212	26.5	96	4.8
	Very poor	58	7.2	17	0.9

Table DS5 Distribution of scores – ReQoL and other measures

	n	mean	standard deviation	completion rate %
ReQoL -10				
Baseline	4037	21.99	10.26	95
Follow-up	953	24.18	10.08	
ReQoL -20 (scale 0 to 80)				95
Baseline	4037	43.27	19.93	
Follow-up	953	48.56	19.57	
ReQoL -20 (scale 0 to 40)				95
Baseline	4037	21.63	9.97	
Follow-up	953	24.28	9.78	
SWEMWBS total				
Baseline	1103	23.14	6.80	95
Follow-up		24.35	6.43	
SWEMWBS rasch				
Baseline	1103	21.71	5.85	95
Follow-up		22.64	5.66	
EQ-5D				
Baseline	1592	0.75	0.25	98
Follow-up		0.78	0.22	
CORE-10				98
Baseline	216	17.79	10.94	
Follow-up	46	16.34	10.57	
PHQ-9				
Baseline	690	13.12	7.74	89
Follow-up		12.39	6.96	
GAD-7				
Baseline	554	6.24	5.18	96
Follow-up		12.08	7.44	

Note: ReQoL -10 in its embedded form of 40 items

Figure DS2a: Distribution of ReQoL-10 scores at baseline

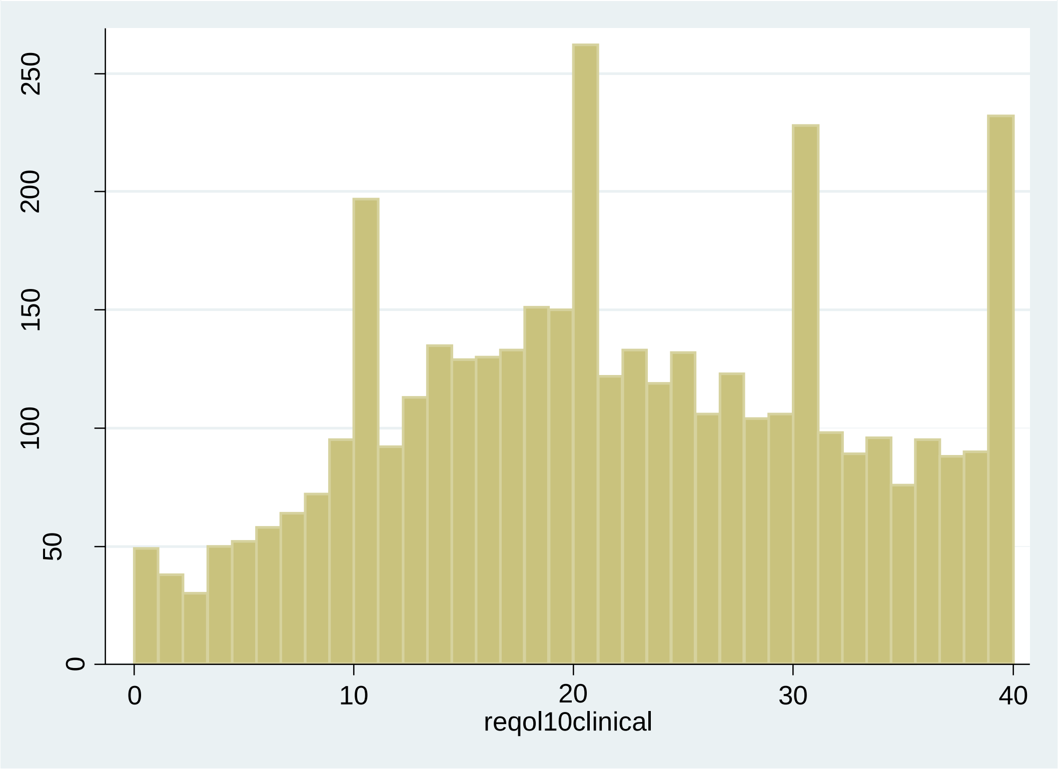

Figure DS2b: Distribution of ReQoL-20 scores at baseline on a scale 0 to 80

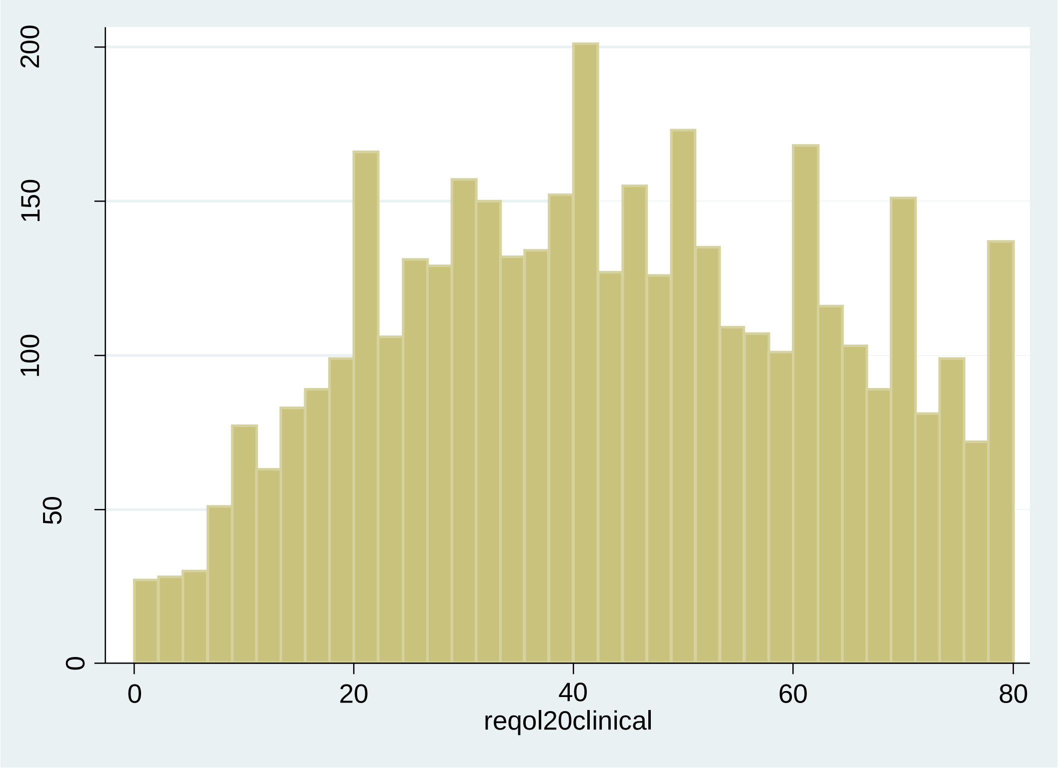

Convergent validity: Lowess scatter plots between ReQoL-10 and the other measures

Figure DS3a: Lowess scatter plots between ReQoL-10 and ReQoL-20 (scale 0 to 40) at baseline

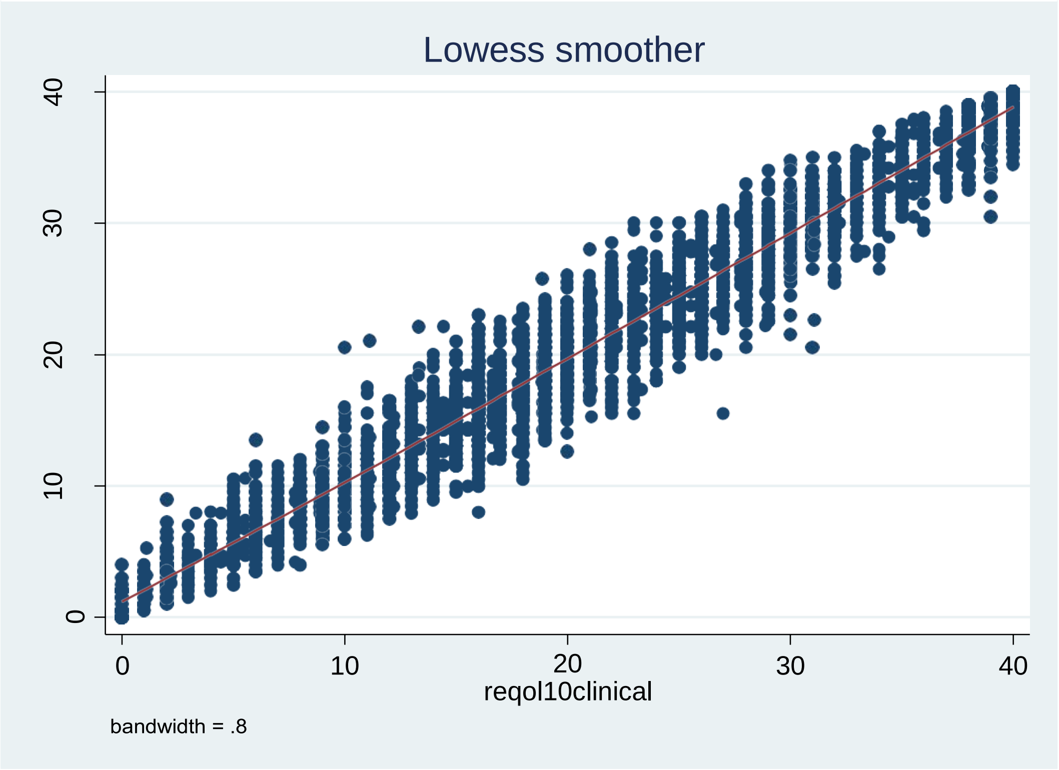

Figure DS3b: Lowess scatter plots between ReQoL-10 and SWEMWBS total score at baseline

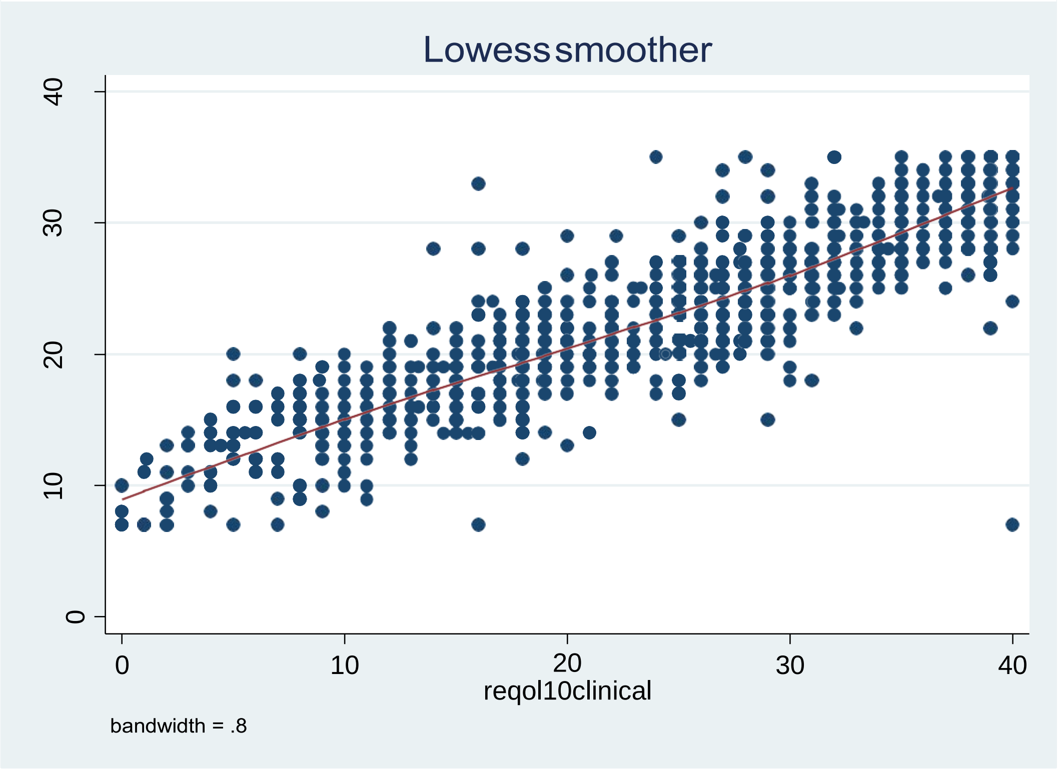

Figure DS3c: Lowess scatter plots between ReQoL-10 and SWEMWBS Rasch score at baseline

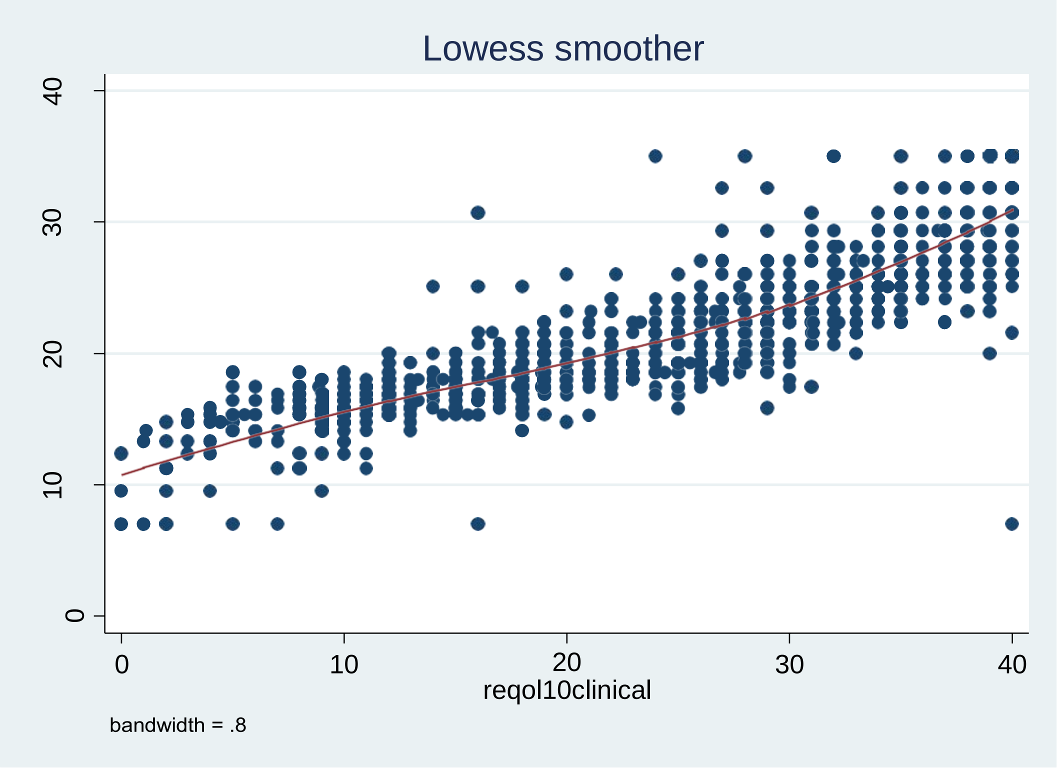

Figure DS3d: Lowess scatter plots between ReQoL-10 and CORE-10 at baseline

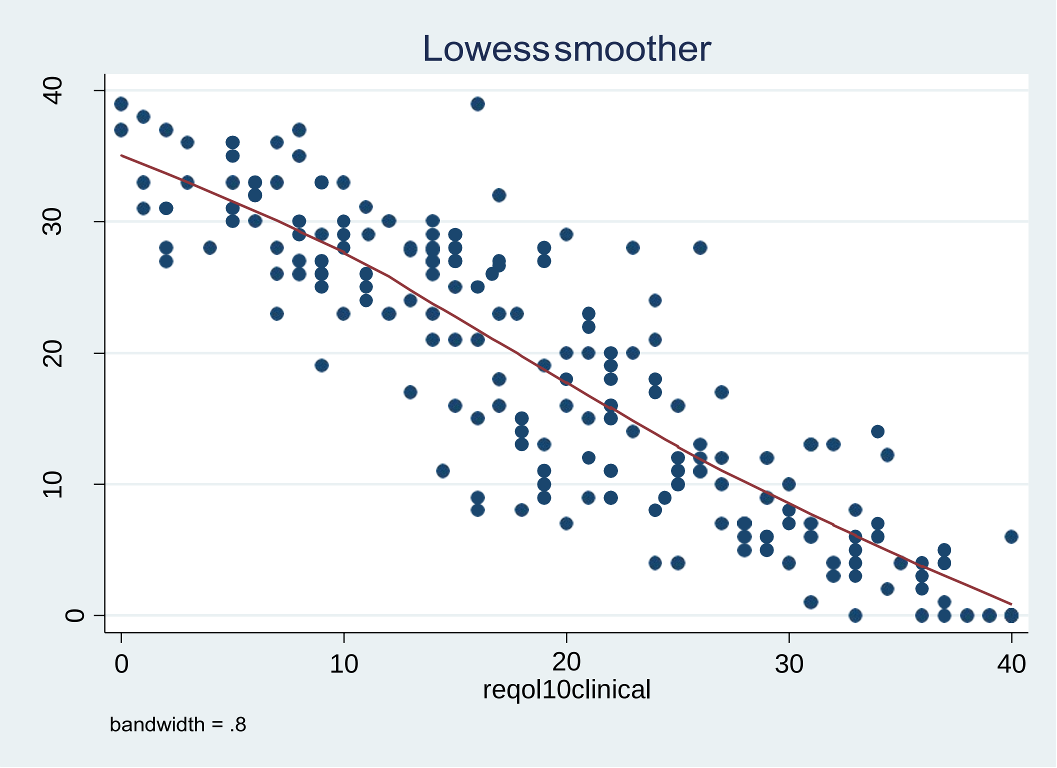

Table DS6a: Known group validity: comparing ReQoL-10 and EQ-5D

	ReQoL-10				EQ-5D			
	n	mean(sd)	p value	SES	n	mean(sd)	p value	SES
General population v patient population	1671<br><br>1513	28.48 (6.96)<br><br>24.02 (10.04)	<0.001	0.64	996<br><br>1513	0.88 (0.21)<br><br>0.75 (0.25)	<0.001	0.59
**Comparing general population and the main disease areas**								
Common mental health disorders	530	22.10 (9.61)	<0.001	0.92	530	0.73 (0.25)	<0.001	0.68
Psychotic disorders	190	24.61 (9.40)	<0.001	0.56	190	0.78 (0.23)	<0.001	0.44
Bipolar	97	23.13 (9.47)	<0.001	0.77	97	0.74 (0.26)	<0.001	0.64
Personality disorder	59	15.71 (8.47)	<0.001	1.83	59	0.63 (0.27)	<0.001	1.15
Other MH disorders	89	20.82 (9.96)	<0.001	1.10	89	0.71 (0.26)	<0.001	0.78
Using self-reported global assessment of health (Good versus Poor)	893<br><br>572	27.62 (8.90)<br><br>18.47 (9.12)	<0.001	1.03	893<br><br>572	0.87 (0.13)<br><br>0.57 (0.27)	<0.001	2.31
Using self-reported global assessment of **mental** health (Good versus Poor)	1151<br><br>321	27.44 (8.12)<br><br>12.00 (6.39)	<0.001	1.90	1151<br><br>321	0.82 (0.19)<br><br>0.51 (0.28)	<0.001	1.63

Table DS6b: Known group validity: comparing ReQoL-10 and SWEMWBS transformed (rasch) score

	ReQoL-10				SWEMWBS transformed			
	n	mean(sd)	SES	p value	n	mean(sd)	SES	p value
General population v patient population	1671<br>1007	28.48 (6.96)<br>24.61 (10.62)	0.56	<0.001	7196<br>1007	23.61 (3.9)<br>21.73 (5.86)	0.48	<0.001
**Comparing general population and the main disease areas**								
Common mental health disorders	371	23.38 (10.09)	0.78	<0.001	371	20.73 (4.71)	0.74	<0.001
Psychotic disorders	52	23.20 (9.52)	0.76	<0.001	52	21.15 (5.59)	0.63	<0.001
Bipolar	98	22.12 (10.31)	0.91	<0.001	98	20.70 (5.83)	0.75	<0.001
Personality disorder	46	13.93 (8.41)	2.09	<0.001	46	16.45 (4.70)	1.84	<0.001
Other disorders	n low							
Using self-reported global assessment of health (Good versus Poor)	583<br>379	28.55 (9.34)<br>18.54 (9.44)	1.07	<0.001	583<br>379	23.71 (5.67)<br>18.62 (4.46)	0.90	<0.001
Using self-reported global assessment of **mental** health (Good versus Poor)	751<br>205	28.19 (8.60)<br>12.42 (6.91)	1.83	<0.001	751<br>205	23.38 (5.32)<br>16.25 (3.48)	1.34	<0.001

Table DS6c: Known group validity: comparing ReQoL-10 and SWEMWBS total (summative) score

	ReQoL-10				SWEMWBS total			
	n	mean(sd)	SES	p value	n	mean(sd)	SES	p value
General population v patient population	1671<br><br>1007	28.48 (6.96)<br><br>24.61 (10.62)	0.56	<0.001	7196<br><br>1007	25.30 (4.72)<br><br>23.25 (6.81)	0.43	<0.001
Comparing general population and the main disease areas								
Common mental health disorders	371	23.38 (10.09)	0.78	<0.001	371	22.19 (5.89)	0.66	<0.001
Psychotic disorders	52	23.20 (9.52)	0.76	<0.001	52	22.31 (6.51)	0.63	<0.001
Bipolar	98	22.12 (10.31)	0.91	<0.001	98	21.74 (6.83)	0.75	<0.001
Personality disorder	46	13.93 (8.41)	2.09	<0.001	46	16.4 (5.82)	1.89	<0.001
Other disorders	n low							
Using self-reported global assessment of health (Good versus Poor)	583<br><br>379	28.55 (9.34)<br><br>18.54 (9.44)	1.07	<0.001	583<br><br>379	25.56 (6.14)<br><br>19.44 (5.87)	1.00	<0.001
Using self-reported global assessment of **mental** health (Good versus Poor)	751<br><br>205	28.19 (8.60)<br><br>12.42 (6.91)	1.83	<0.001	751<br><br>205	25.29 (5.74)<br><br>16.00 (4.60)	1.62	<0.001

All the correlation coefficients are significant at 1%

Table DS7: Floor and ceiling effects at baseline and follow-up

	% at worst score		% best score	
	T1	T2	T1	T2
ReQoL-10	0.72	0.63	3.77	4.6
ReQoL-20	0.30	0.32	1.49	1.9
SWEMWBS Total score	1.52	1.06	4.67	4.6
SWEMWBS Rasch score	1.52	1.06	4.67	4.6
EQ-5D	0.00	0.00	14.04	15.7
